# Profiles of genetic parameters of body weight and feed efficiency in two divergent broiler lines for meat ultimate pH

**DOI:** 10.1186/s12863-022-01035-z

**Published:** 2022-03-16

**Authors:** Quentin Berger, Elodie Guettier, Jérémy Bernard, Patrice Ganier, Marine Chahnamian, Elisabeth Le Bihan-Duval, Sandrine Mignon-Grasteau

**Affiliations:** 1 INRAE, Université de Tours, BOA, 37380 Nouzilly, France; 2 INRAE, PEAT, 37380 Nouzilly, France

**Keywords:** Kinetics, Growth, Feed conversion ratio, Genetic determinism, Chicken, Alternative feedstuff, Meat quality

## Abstract

**Background:**

Selection for feed efficiency is one of the best ways to decrease poultry production costs and environmental impacts. While literature on its genetic determinism is abundant, it is limited to one or a few periodic values over the animals’ lifespans. With the development of new phenotyping tools, kinetics of growth and feed intake are now available, providing access to daily data on feed efficiency. In this study, over the course of 6 weeks, we described the kinetics of body weight (BW), average daily weight gain (ADG), feed intake (FI), and daily cumulative feed conversion ratio (DCFCR) using electronic feed stations. We then estimated the genetic parameters of daily data in two fast growing lines of chicken divergently selected for breast meat ultimate pH (heritability and genetic correlations with breast meat yield and pH).

**Results:**

Birds from the line selected to have a more acidic meat (pHu-), were more efficient than those selected to have a less acidic meat (pHu+), with a 4.3% higher BW from d7 to d29 and 5.0% better feed efficiency from d12 to slaughter. The line effect for ADG and DCFCR appeared to be significant as early as d5, which is consistent with the early age at which metabolic differences between the two lines appear. Genetic parameters estimated within each line revealed different genetic determinisms of growth and feed efficiency, with a higher impact of maternal effects on BW during the growing phase (d10 to d20) in the pHu+ line and much higher heritability values of DCFCR during the finishing phase (d26-d42) in the pHu- line.

**Conclusion:**

Genetic profiles of daily performance highlighted the difference between both lines. Their behavior during dietary transitions reinforced the already known impact of these periods in the animals’ lives. Based on the profiles of genetic parameters within each line, it seems feasible to identify early criteria for selecting feed efficiency, but they must be defined for each line, as the genetic determinism of these traits is line-dependent.

**Supplementary Information:**

The online version contains supplementary material available at 10.1186/s12863-022-01035-z.

## Background

Feed efficiency has been one of the most important criteria of selection in poultry for decades because of its economic importance and benefits for the environmental impact of poultry production [1]. In the US context, with an annual production of 19.5 10^9^ tons of poultry meat, Abasht et al. [2] estimated that a 1% reduction in feed conversion ratio would result in a savings of $ 294 10^6^ on broiler feed and a reduction of 0.53 10^6^ tons of fresh manure produced. Diversifying feedstuffs is another way to reduce the social impact of poultry production by increasing the proportion of local or less concentrated feedstuffs and agricultural by-products in poultry diets, and reducing importations of soybean into Europe [3] and competition between human and animals for lands and cereals [4–6].

Literature on the genetic determinism of feed efficiency is abundant. Estimates of heritability vary largely depending on the periods studied and the strains. For example, heritability estimates for feed conversion ratio at 42 d vary from 0.13 to 0.41 depending on the strains and on the duration of data collection [4, 7–10]. Estimates are generally lower in commercial lines than in random-bred or crossbred lines. Data from the literature also indicate that heritability of feed conversion ratio tends to decrease with age [2]. Despite this obvious effect of age, most of the literature on the genetics of feed efficiency has been concentrated on the last part of the production cycle, has relied on one or a few data of feed efficiency calculated over periods of one to 6 weeks, and no daily genetic parameters are available in the literature yet. Finally, despite the fact that all meat-type chickens and an increasing proportion of laying hens are reared in group and on floor, most estimates of genetic parameters of feed efficiency rely on measures of feed intake collected in individual cages.

Thanks to recent developments in the phenotyping of feed consumption and animal weight, it is now possible to have access to the kinetics of animal growth and feed efficiency. Thus, using the BIRD-e electronic feeder that provides daily data of feed intake and body weight on individuals raised on the floor [11, 12], Berger et al. [4] described the response of slow- or fast-growing chickens to conventional or alternative diets. Their work highlighted several interesting points such as the importance of transition periods around diet changes and the impact of early growth or feed efficiency parameters on the final performance of birds, including body composition and meat quality.

These new phenotyping tools make it possible to revisit the genetic study of feed efficiency in chickens and to optimize the search for selection criteria by considering the kinetics of daily data. The objective of this study was thus to describe the evolution profile of genetic parameters of body weight, feed intake and feed conversion ratio in fast-growing chickens, in a context of feedstuffs diversification. Animals were thus fed with a diet containing the same amount of energy and proteins than usual diets, but including alternative feedstuffs to soybean, produced locally and less in competition with human food. Feed efficiency depends on many factors such as basal metabolism, protein turnover, body composition, and digestive efficiency [2]. A link with meat quality traits such as wooden breast defect was also recently reported [13]. In the current study, we relied on two lines of fast-growing broilers divergently selected for breast meat ultimate pH [14], in order to study the genetic control of the kinetics of body weight and feed efficiency in birds with different metabolic profiles and meat quality.

## Results

### Line effect on phenotypic performance

As expected from the selection performed on these divergent lines, breast meat ultimate pH at 42 d was much lower in the pHu- line than in the pHu+ line (5.58±0.12 vs 6.21±0.14, *P*= 0.001). Breast meat yield (BMY) was also 3.5% lower in the pHu- line than in the pHu+ line (18.83±1.54 vs 19.51±1.82, *P*=0.001).

**Fig. 1:**
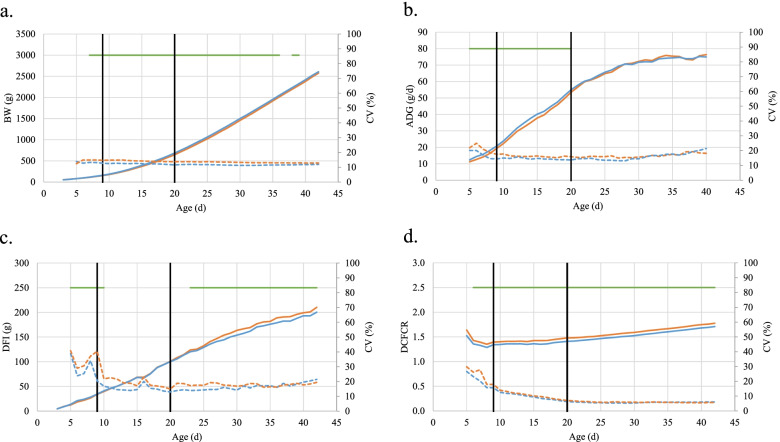
Kinetics of BW (**a**), ADG (**b**), DFI (**c**) and DCFCR (**d**) in both chicken lines. Solid lines are used for mean values, dotted lines for coefficient of variation. Orange line stands for pHu+ line, blue line stands for pHu- line. Black vertical lines are indicating diet changes. Green horizontal lines are indicating the period of significance of the line effect

The line effect on daily performance is shown in Fig. 1 and detailed statistics can be found in Supplementary Tables 1 and 2. Line effect was significant from 8 to 36 days for BW, from 5 to 20 days for ADG, from 5 to 10 days and from 23 to 42 days for DFI, and from 6 to 42 days for DCFCR. During these periods, pHu- birds showed better performance than those from the pHu+ line (7.3% higher ADG, 4.3% higher BW, and 4.2% lower DCFCR). The ranking of both lines changed for DFI between the first period (5-10 d), during which pHu+ birds had a 9.1% lower feed intake, and the second period (23-42 d) during which pHu- birds ate 4.3% less than pHu+ birds.

For all daily traits, pHu+ performance was more variable than pHu- performance, as shown by larger coefficients of variation reported in Fig. 1. For growth traits, the performance variability of both lines was constant throughout the entire period (with CV comprised between 11 and 25%), while the variability of feed intake and feed efficiency was much higher before the first change of diet (CV from 15.7 to 38.8%) than after (CV from 5.3 to 21%).

### Heritability estimates for daily performance and slaughter traits

Heritability of pHu and BMY were quite significant and close in the two lines (pHu: 0.55±0.03 in pHu- and 0.58±0.03 in pHu+, BMY: 0.59±0.03 in pHu+ and 0.54±0.03 in pHu-).

**Fig. 2:**
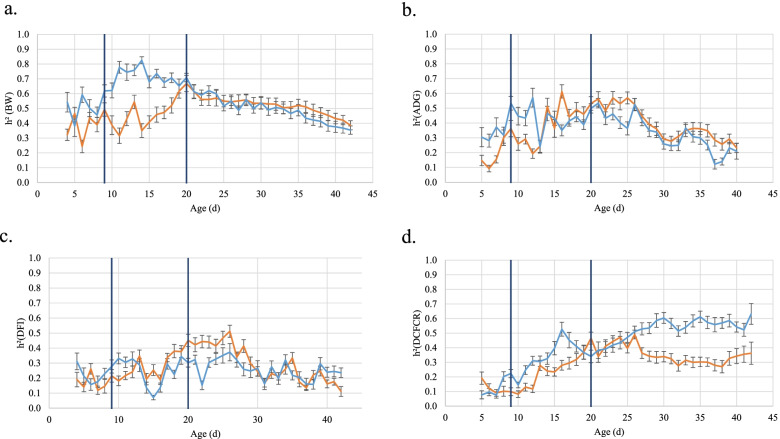
Heritability estimates Heritability estimates (±1 standard error) of (**a**) BW, (**b**) ADG, (**c**) DFI, and (**d**) DCFCR. Orange line stands for the pHu+ line and blue line for the pHu- line. Black vertical lines are indicating diet changes

Heritability estimates of daily performance are presented in Fig. 2 and supplementary Table 3. Heritability of DCFCR (Fig. 2d) increased with age for both lines, until 26 days for pHu+ and 30 days for pHu-. Then it remained stable around 0.57 for the pHu- line and slightly decreased to stabilize around 0.30-0.35 for the pHu+ line.

Heritability of ADG and DFI showed comparable kinetics (Figs. 2b, c). Before 26 d, heritability tended to increase in the pHu+ line whereas it was stable in the pHu- line. After 26 d, heritability estimates decreased in both lines for ADG. For DFI, it decreased to 0.11 for pHu+ whereas it remained stable around 0.25 for the heritability of DFI for pHu-. The largest difference in heritability between the two lines for both traits occurred on the 8 days after the second change of diet.

Finally, for BW, heritability increased until 14 d for pHu- and 20 d for the pHu+ line, estimates being 14 to 146% higher for pHu- (Fig. 2a). After this first age, heritability estimates from both lines decreased until 42 d and remained close between the two lines, with slightly higher values after 35 days in the pHu+ line (9 to 13%).

### Maternal effects on daily performance

**Fig. 3:**
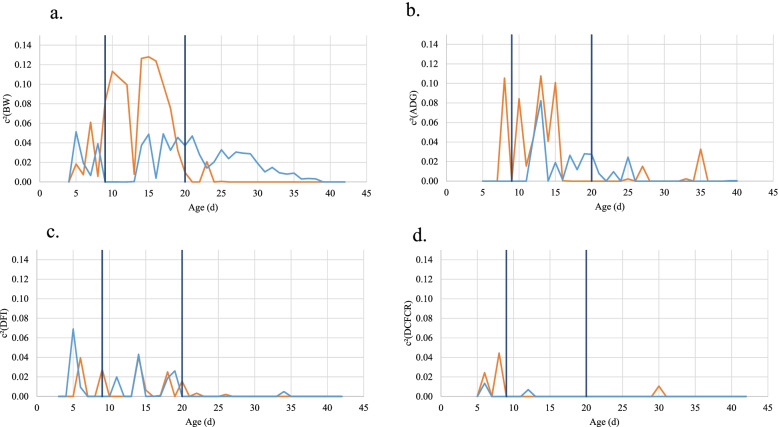
Maternal permanent environment effect (c^2^) on (**a**) BW, (**b**) ADG, (**c**) DFI, and (**d**) DCFCR. Orange line stands for the pHu+ line and blue line for the pHu- line. Black vertical lines are indicating diet changes

Maternal effects did not exceed 0.07 for both lines for DCFCR and DFI and nearly disappeared after the first diet change for DCFCR and the second diet change for DFI (Figs. 3c, d). In contrast, maternal effects reached higher values and remained present later for BW and ADG (Figs. 3a, b), especially in the pHu+ line. For both traits, maximum values were reached for pHu+ animals between the two diet changes (0.10 for ADG and 0.13 for BW). Maternal effects on BW in pHu- were lower (<0.08), but remained significant until 38 d.

### Genetic correlations

#### Correlations between final cumulative feed conversion ratio and daily performance

**Fig. 4:**
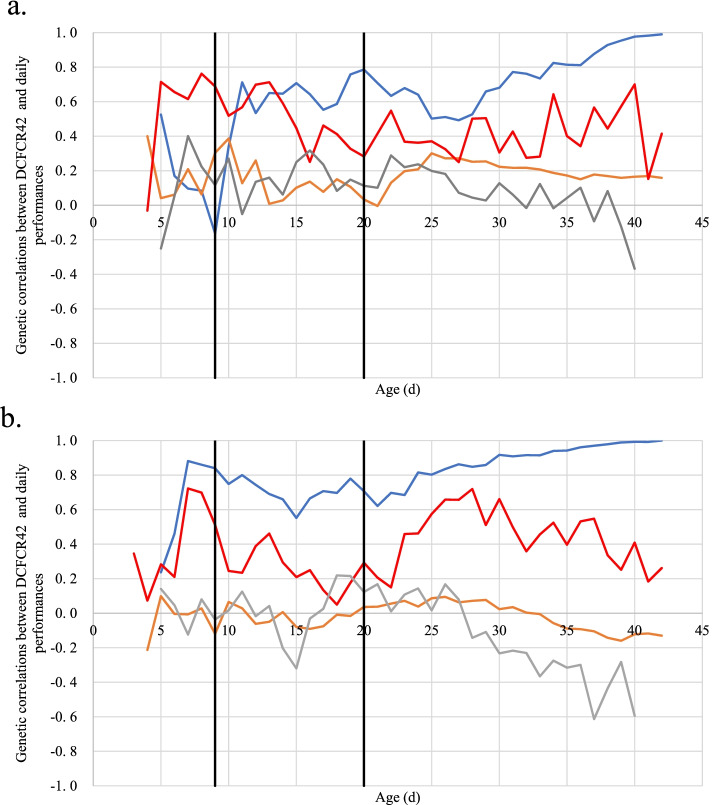
Kinetics of genetic correlations between (DCFCR_42_) and daily performance for pHu+ (**a**) and pHu- (**b**). DCFCR_42_ is for final cumulative feed conversion ratio. Correlations with daily cumulative feed conversion ratio are in blue, with body weight in orange, with daily feed intake in red, with average daily gain in grey. Black vertical lines represent diet changes

Genetic correlations between final cumulative feed conversion ratio (DCFCR_42_) and daily traits are reported in Fig. 4 and Supplementary Table 4. As expected, the genetic correlation between daily and final cumulative FCR increased with age. It reached strong values before the first diet change in pHu- (>0.8) and just after in pHu+ (>0.7), suggesting that feed efficiency recorded around the first diet change is an early predictor of final feed efficiency. In contrast, correlations between DCFCR_42_ and body weight were low throughout the whole period in both lines (0.17 in pHu+ and -0.02 in pHu- on average).

Profiles of correlations between DFI and DCFCR_42_ varied more between the two lines. Two periods of stability occurred for pHu+. The first one occurred between day 5 and day 13 and showed values around 0.62. The second one was between day 15 and slaughter, and showed values around 0.4. The correlation between DFI and DCFCR_42_ in pHu- was more variable, increasing to 0.7 before the first change of diet and decreasing to 0.2 the day after. After this change, the correlation remained stable until 22 days. Then it increased strongly to 0.72 at 29 days and finally decreased to 0.2 in the last days. The line difference could also be seen in the correlations between DCFCR_42_ and ADG. Thus, the correlation within the pHu+ line remained stable and low during the whole life of the chickens (0.10 on average), whereas this correlation showed a decrease after 27 days in the pHu- line. Average correlation values were equal to 0.04 before 27 d and -0.32 after 27 d, indicating that in pHu- a lower efficiency was associated with a decreased growth rate at later ages.

### Correlation between pHu and daily performance

**Fig. 5:**
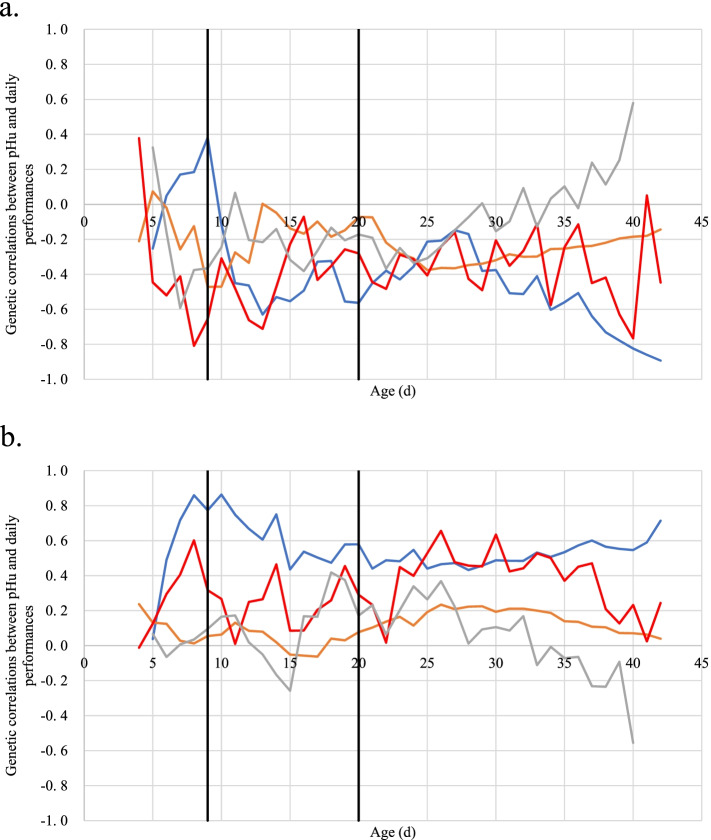
Kinetics of genetic correlations between pHu and daily performance for pHu+ (**a**) and pHu- (**b**). Correlations with the DCFCR are in blue, with BW in orange, with DFI in red, and with ADG in grey. Black vertical lines represent diet changes

Genetic correlations between the criteria of selection, breast meat ultimate pH, and daily traits are reported in Fig. 5 and Supplementary Table 5. Since the lines had been divergently selected for pHu, different patterns of correlations between pHu and growth or feed efficiency traits could be expected between the two lines. Indeed, for most correlations, the signs of correlations were different in the two lines. For example, correlations between BW or DFI and pHu were low and negative in pHu+ (μ=-0.23 and -0.37, respectively), but low and positive in pHu- (μ=+0.11 and +0.33, respectively). Regarding the correlation between ADG and pHu, the difference of pattern between lines was marked after the second diet change, as this correlation increased from -0.24 to +0.58 in pHu+, but decreased from +0.37 to -0.56 in pHu-.

The correlation between DCFCR and pHu increased in both lines from hatch to the first diet change (to +0.38 for pHu+ and +0.80 for pHu-). After this event, the correlation for pHu+ decreased down to -0.6 at 13 days and remained stable until the second diet change. Finally, the correlation increased slowly up to -0.15 at 28 days. From 29 d until the end, it decreased to reach -0.89. The correlation for pHu- was more stable, decreasing after the first change of diet and being stable from 15 to 30 days, at around +0.53. After this day, it increased and reached +0.71 at 42 days.

### Correlation between BMY and DCFCR

**Fig. 6:**
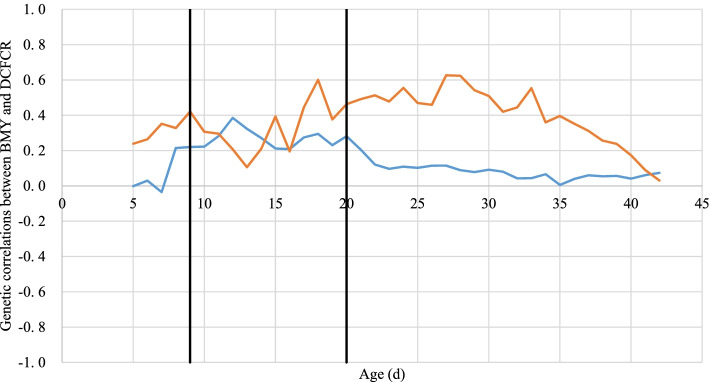
Kinetics of genetic correlations between BMY and the DCFCR for both lines. Correlation for pHu+ are in orange and for pHu- in blue. Black vertical lines represent diet changes

Figure 6 and Supplementary Table 6 contain the genetic correlations between DCFCR and BMY. For the pHu- birds, this correlation first increased from 0 to 0.38 until 12 d and then decreased until 42 d, being close to 0 at the end. In comparison, the correlation for pHu+ showed 3 phases: from 0 to 16 d around 0.2-0.4, from 17 to 28 d between 0.4 and 0.6, and from 29 d to slaughter a decrease until 0.

## Discussion

This study made it possible to give a detailed description of the kinetics of growth and feed efficiency in the two divergent lines for breast meat ultimate pH. While the weight of chicks was higher at hatching in the pHu+ line (40.9 vs 38.4, P<0.001), their daily feed intake was lower in the first days of life so that BW became similar between the two lines at 5 d. As ultimate pH and muscle glycogen stores are highly genetically correlated at slaughter age (-0.97) [15], selection applied to pHu modified glycogen levels between pHu+ and pHu- broiler chickens [14, 16]. This difference was found to be already present at hatch, with a higher glycogen content in the breast and pipping muscles of pHu- chicks [17]. These early metabolic differences are accompanied by a lower quality of pHu+ chicks [18] estimated according to the Tona’s grid [19]. In poor quality chicks, the starting phase seems more difficult with an altered feed intake in the first days of life and a lower average daily gain continuing up to 3 weeks of age, as evidenced in the current study. As shown by transcriptomic and metabolomic analyses conducted at 6 weeks [20, 21], growing chickens from the two lines rely on different metabolic pathways to produce energy, with an intensive use of carbohydrate metabolism in the pHu- line and of muscle proteolysis and amino acid catabolism as well as lipid oxidation in the pHu+ line. In the current study, we showed that although the pHu+ birds consumed a higher amount of feed from 23 days onwards, they were characterized by a lower BW and 4% higher FCR, in consistency with their lower metabolic efficiency. At the same time, pHu+ birds had a higher breast yield at slaughter (19.5 vs 18.8, P<0.001). Although a favorable relationship is usually observed between breast development and feed efficiency [1], this was not observed in the pHu lines for which the higher protein deposition in the pHu+ line seems to be made at a higher energetic cost. This absence of correlation and the lower DCFCR heritability in pHu+ birds after the second change of diet may underline a difficulty for these animals to express fully their genetic potential. This could be due to the fact that the energetic needs are fulfilled by the diet for the pHu- birds but not for the less efficient pHu+ birds. For example, the Hubbard nutritional recommendations during the finishing phase vary between 2850 and 3200 kcal/kg [22]. Our diet is formulated at 2950 kcal/kg, which may be sufficient in pHu- but not in pHu+, whose needs may be closer to the upper values recommended. During this period, this lack of energy is compensated by an increased feed intake, leading to an identical growth in the two lines but to a higher DCFCR in pHu+ line. We can thus hypothesize that a diet higher in energy would be more appropriate in the pHu+ line.”

The current study also provided original results on the genetic determinism of the kinetics of growth and feed efficiency in chickens. While heritability of BW evolved in a similar way in the two lines from 20 days onwards, decreasing from high values of 0.7 to moderate values of 0.35-0.40, genetic controls of BW differed between the two lines in the first part of life. In particular, BW heritability was much lower between 10 d and 20 d in the pHu+ line. This may partly be due to maternal effects that greatly influenced BW variability during this period in the pHu+ line, while they remained low in the pHu- line. In the current standardized conditions of poultry production, maternal effects are mainly mediated by egg characteristics. Factors such as egg size and nutrient content (vitamins, carotenoids, etc.) or maternal hormones can impact the development and the phenotypes linked to the fitness of the offspring [23]. The higher egg weight observed in the pHu+ line [17] might contribute to the higher maternal effects observed in this line, but additional studies are needed to evaluate if variations in nutrient and hormone contents in the eggs could also exist between the two lines.

The range of estimates of heritability of feed conversion ratio available in the literature was 0.11 to 0.54 [2, 7, 24–29]. These values are obtained between 3 and 6 weeks of age, as animals in these studies are most often reared on floor several weeks and transferred to individual cages during 1 to 3-4 weeks for the measure of feed intake. Estimates of heritability of feed efficiency before 2 weeks are thus not available in the literature. Our values obtained at similar ages, i.e. after the second diet change, are on the top of this range, with average estimates of 0.35 in pHu- line and 0.53 in pHu+ line. Moreover, at the exception of Howie et al. [28], these estimates were obtained in animals reared in individual cages, where social behavior and physical activity which influence feed intake and growth, cannot be expressed normally. Our estimates are quite close to those of Howie et al. [28] obtained on floor- and group-reared animals, which varied from 0.30 to 0.45 in four different genotypes for the global feed conversion ratio between 2 and 5 weeks. However, even in the latter case, the absence of daily measure of body weight prevented calculating daily feed conversion ratio, and thus following its evolution with animal growth. We show in this paper that this daily evolution is large, as heritability of feed conversion ratio varies strongly between early ages (0.12-0.14 on average before the first diet change) and late ages (0.35-0.53 on average after the second diet change). In contrast to BW, the genetic difference between the two lines for feed efficiency was mostly present after 25 days, when heritability stabilized at a high value of 0.5-0.6 in the pHu- line and at a moderate value of 0.30 in the pHu+ line. A difference in the profiles of heritability of feed efficiency around diet transitions was also evidenced. Indeed, pHu+ heritability was quite stable around these periods, whereas pHu- showed a strong increase in heritability around the first transition and a sharp decrease before the second transition. The profiles of genetic correlations showed that, in both lines, maintenance costs were not decisive for feed efficiency, but that feed intake was the trait contributing the most to feed efficiency. However, after 30 d in the pHu- line, the influence of feed intake decreased in favor of weight gain. The difference in energetic metabolism between lines may contribute to these differences in genetic profiles. At the first transition, feed intake and body weight both increased linearly and needs may be fulfilled thanks to the increase of the ratio of energy to protein between the first and the second diet. In contrast, between 15 and 20 days, feed intake increase was still linear whereas growth was exponential. For pHu- birds, the ratio of energy to protein in the second diet may thus be limiting to fulfill animal needs until the last diet transition, after which heritability increased again and reached a higher value than in the pHu+ line.

Access to daily data can offer new opportunities to improve selection strategies for feed efficiency. At first, the profiles of genetic parameters can lead to identifying earlier predictors of the final feed efficiency. By combining information on heritability and genetic correlations, we compared expected response to a direct selection for the cumulative feed conversion ratio measured at slaughter age (i.e. DCFCR_42_) to the indirect selection for the same indicator measured earlier. Given our experimental results, selection for DCFCR could be envisaged as soon as 20 d in pHu+ birds and 29 d in pHu- birds with an expected response amounting to 80% of the maximum response that would be obtained by selecting for DCFCR_42_. Whatever the scenario, the expected response would be higher in the pHu- line than in the pHu+ line (0.42±0.14 and 0.21±0.09, respectively for pHu- and pHu+). Moreover, given the profiles of correlations with BMY and meat pHu, improved FE would lead to an increased pH value and a decreased breast yield in the pHu+ line, but a more acidic meat without any correlated response on BMY in the pHu- line. This illustrated again the different genetic determinism of FE in the two lines.

Selection on feed conversion ratio with an alternative diet would improve the efficiency of the system by two ways 1) the improvement of FCR performance with a high economic impact or 2) at the same FCR, the spare of resources that could be used in human food. In a former study, we already showed that rapid growing chickens could reach similar performances with a classical corn-soybean diet than with an alternative diet [1]. We considered the proportion of energy and protein of each feedstuff that could be used for human food [30], the composition of our alternative diet and of the classical diet as used in Berger et al. [1], and the mean feed intake of starter, grower and finisher diets we got in the current study to calculate the quantity of human-edible energy and proteins that could be spared by using an alternative diet instead of a classical diet. For each 2 kg chicken fed with the alternative diet and with the same FCR, depending on the line, we would save 63.8-65.2 g of human-edible proteins and 153.5-158.0 g of human-edible energy than with the classical diet. This represents a decrease of 1.4% on protein and 1.8 to 3.5% on energy. This saving of resources by changing diet composition without any FCR improvement is roughly equivalent to the gain expected by a genetic improvement on the alternative diet of 0.10 of FCR for human-edible proteins (77.8-80.2 g) and by a decrease of 0.15 of FCR for human-edible energy (155.4-159.7 g).

## Conclusion

Our study highlighted differences in the kinetics of feed intake, growth rate, and feed efficiency between the two broiler lines diverging for breast meat ultimate pH in relationship with their early metabolic differences. The genetic determinism of these traits varied to some extent between the two lines, and showed that the respective contributions of growth rate and feed intake on genetic determinism of FCR differed in the two metabolic contexts. Having access to daily FCR data allowed for the identification of early relevant predictors, and opens the way to new strategies or methodologies for selecting feed efficiency.

## Materials and methods

### Birds and housing

In this study, we used animals from two experimental broiler lines divergently selected for a high (pHu+) or a low (pHu-) ultimate pH of the *Pectoralis major* muscle during fourteen generations. These lines came from a fast-growing grandparental female broiler line selected for both growth and reproduction traits. A complete description of the breeding scheme and performance of these lines can be found in Alnahhas et al. [14]. Two batches were reared in parallel for 42 days for the current study, the second batch starting one week later than the first one. Each batch was reared in identical and adjacent cells of 11 m × 5.11 m covered with wood shavings. The first and second batches respectively included 183 and 156 pHu- and 144 and 167 pHu+ of both sexes.

The temperature was set at 32°C at hatch and decreased progressively to reach 20-23°C from 29 d on. The light program was 24D:0N at d0, 23D:1N at 1 d and 18D:6N from 3 to 42 d. At hatch, the animals were identified with a wing band and an electronic Radio frequency identification device (RFID) chip, then weighed and placed in one pen on a floor covered with wooden chips. The RFID chip was placed at the base of the neck and secured with a plastic string passing under the skin.

The animals were fed *ad libitum* with a diet containing a high proportion of sunflower, rapeseed, and fava bean in order to reduce the soybean meal proportion in the diet. The detailed composition is shown in Table 1. A starter diet was given from hatch to 8 d (2850 kcal.kg^-1^ DM; 21.5 % CP), a grower diet from 9 to 19 d (2900 kcal.kg^-1^ DM; 20.0 % CP), and a finisher diet from 20 to 42 d (2950 kcal.kg^-1^ DM; 18.5 % CP). Diet was formulated using the PORFAL software v.4.0.17 (ITP-INRA, Paris) in order to follow Hubbard broiler nutrition recommendations [22]. Potential interesting feedstuffs, such as Faba bean, sunflower meal, rapeseed and DDGS were included in the diet based on the results of a former project dedicated to the test of alternative feedstuffs for poultry diets [31].

**Table 1:** Composition and age of distribution of diets

Ingredient (%)	1-8 d	9-20 d	21-42 d
Corn	20.42	19.89	23.50
Wheat	30.10	30.10	30.10
Fava bean	-	12.00	13.00
Soybean meal	24.22	11.61	7.13
Rapeseed meal	5.00	5.00	8.00
Wheat DDGS	3.00	5.00	5.00
High fiber sunflower meal	8.12	7.73	5.19
Soybean oil	5.00	5.00	5.00
Corn gluten			
Calcium carbonate	0.655	0.142	0.00
Bicalcic phosphate	2.05	1.73	1.40
Salt	0.192	0.15	0.158
Vitamins and minerals	0.40	0.40	0.40
Sodium carbonate	0.196	0.262	0.250
DL-Methionine	0.234	0.285	0.234
HCL Lysine	0.176	0.264	0.250
Threonine	0.088	0.157	0.135
Valine	0.038	0.130	0.106
Tryptophane	-	-	0.005
**Calculated composition**			
AMEn, kcal/kg	2850	2900	2950
CP, g/kg	215	194	181
Lys, g/kg	11.2	10.9	10
Met + Cys, g/kg	8.4	8.17	7.5
Trp, g/kg	2.28	1.84	1.7

Body weight and feed intake were individually and continuously recorded throughout the experiment thanks to electronic feed stations [11] and calculated following the procedure presented in Berger et al. [4].

### Body weight, feed intake, weight gain, and feed conversion ratio

Body weight (BW), daily feed intake (DFI), average daily gain (ADG), and daily cumulative feed conversion ratio (DCFCR) were calculated as in Berger et al. [1]. Briefly, for each animal i at day j, average daily gain (ADG_ij_) and daily cumulative feed conversion ratio (DCFCR_ij_) were calculated as:

$${ADG}_{ij}=\frac{BW_{i\left(j+2\right)}-{BW}_{i\left(j-2\right)}}{5}$$

$$DCFCRij=\frac{\sum_{k=3}^{k=j}{DFI}_{ik}}{BW_{ij}-{BW}_{i3}}$$

where DFI_ij_ and BW_ij_ are the daily feed intake and body weight of animal i for day j, respectively. DCFCR_42_ stands for the cumulative feed conversion ratio over the whole rearing period.

### Meat ultimate pH

At 42 d, the animals were weighed after 8 hours of feed withdrawal and transferred to the slaughterhouse of the PEAT INRAE poultry experimental facility.

After 24 hours of chilling, the right *Pectoralis major* and *Pectoralis minor* were cut and weighed. Breast meat yield (BMY) was calculated as:

$$BMY=100\times \frac{2\times \left( Pectoralis\ major+ Pectoralis\ minor\right)}{BW_{42}}$$

The ultimate pH of the *Pectoralis major* (pHu) was measured with a portable pH meter (model 506, Crison Instruments SA, Alella, Barcelona, Spain).

### Phenotypic analyses

All phenotypes were first analyzed using the PROC GLM procedure of SAS 9.4 [32] with the following model:

$${\mathrm{y}}_{\mathrm{i}\mathrm{jkl}}={\mathrm{L}}_{\mathrm{i}}+{\mathrm{S}}_{\mathrm{j}}+{\mathrm{H}}_{\mathrm{k}}+{\mathrm{e}}_{\mathrm{i}\mathrm{jkl}}$$

with y_ijkl_ being the trait for animal l of line i, sex j and hatch k and e_ijkl_ the residual for animal l.

### Genetic analyses

Heritability and genetic correlations between traits were estimated using VCE6.0 software [33, 34]. In addition to data collected during this experiment, data of pHu collected from previous generations of selection were included in the genetic analyses, leading to a variable number of data depending on the trait: 578 for BW, ADG, DFI, DCFCR and 9,541 for pHu. The pedigree file contained all animals from the beginning of the selection experiment, i.e. 23,319 animals.

Genetic analyses were conducted separately for each line. The animal model included the fixed effects of the sex (*N*=2) and batch (*N*=31) and the direct genetic effect of animal (*N*=23,319). When the maternal permanent environment effect (*N*=1,961) was not null, it was also included in the model of analysis.

## Supplementary Information

**Additional file 1.**

## Data Availability

The datasets supporting the conclusions of this article are available in the Data INRAE repository, under the unique persistent identifier 10.15454/QBNHAD.

## Abbreviations

ADG: Average Daily Gain (g.d^-1^)
BMY: Breast Meat Yield (%)
CP: Crude Protein (%)
CV: Coefficient of Variation (%)
DCFCR: Daily Cumulative Feed Conversion Ratio (g.g^-1^)
DCFCR_42_: Final Cumulative Feed Conversion Ratio at 42 days (g.g^-1^)
DFI: Daily Feed Intake (g.d^-1^)
FCR: Feed Conversion Ratio (g.g^-1^)
FE: Feed Efficiency
pHu: Ultimate pH of breast meat
pHu-: Broiler lines divergently selected for a low pHu
pHu+: Broiler lines divergently selected for a high pHu
RFID: Radio Frequency Identification Device
