# Supplementary material for: Profiles of genetic parameters of body weight and feed efficiency in two divergent broiler lines for meat ultimate pH
Source: BMC Genom Data. 2022 Mar 16;23:18. doi: 10.1186/s12863-022-01035-z (PMC8925093; doi:10.1186/s12863-022-01035-z)
Supplement: Supplementary file 1 — Additional file 1. [file 12863_2022_1035_MOESM1_ESM.docx]

**Supplementary Table 1.** Least square means of daily cumulative feed conversion ratio (DCFCR) and average daily gain (ADG) for the two lines of pHu animals, and significance of the line effect.

|  | DCFCR | | | ADG | | |
| --- | --- | --- | --- | --- | --- | --- |
| Age | pHu- | pHu+ | *P-value* | pHu- | pHu+ | *P-value* |
| 5 | 1.52 | 1.64 | 6.14E-02 | 12.4 | 11.2 | 7.47E-07 |
| 6 | 1.36 | 1.43 | 2.28E-02 | 14.4 | 12.7 | 9.31E-12 |
| 7 | 1.33 | 1.39 | 2.04E-02 | 15.8 | 14.1 | 8.60E-12 |
| 8 | 1.29 | 1.35 | 4.03E-04 | 18.1 | 16.3 | 7.64E-14 |
| 9 | 1.34 | 1.39 | 3.47E-03 | 20.8 | 19.3 | 1.60E-08 |
| 10 | 1.35 | 1.40 | 1.51E-04 | 23.8 | 22.3 | 4.05E-07 |
| 11 | 1.36 | 1.41 | 2.23E-04 | 27.7 | 25.9 | 2.05E-07 |
| 12 | 1.36 | 1.41 | 9.62E-05 | 31.7 | 29.7 | 6.84E-07 |
| 13 | 1.36 | 1.41 | 3.18E-05 | 34.7 | 32.2 | 6.31E-09 |
| 14 | 1.35 | 1.41 | 1.12E-06 | 37.6 | 35.0 | 1.52E-09 |
| 15 | 1.36 | 1.42 | 1.88E-08 | 40.2 | 37.8 | 8.86E-07 |
| 16 | 1.35 | 1.42 | 6.55E-12 | 41.9 | 39.7 | 1.08E-05 |
| 17 | 1.36 | 1.43 | 1.97E-12 | 44.9 | 43.2 | 1.02E-03 |
| 18 | 1.38 | 1.45 | 9.27E-12 | 47.6 | 45.9 | 1.47E-03 |
| 19 | 1.40 | 1.46 | 2.43E-14 | 51.4 | 49.5 | 2.01E-03 |
| 20 | 1.41 | 1.48 | 7.38E-18 | 55.0 | 53.5 | 1.57E-02 |
| 21 | 1.42 | 1.48 | 4.71E-17 | 57.8 | 56.6 | 7.52E-02 |
| 22 | 1.43 | 1.49 | 8.39E-16 | 60.2 | 59.9 | 5.83E-01 |
| 23 | 1.44 | 1.50 | 3.61E-16 | 61.4 | 61.0 | 5.30E-01 |
| 24 | 1.45 | 1.51 | 4.98E-15 | 63.6 | 62.7 | 1.53E-01 |
| 25 | 1.47 | 1.52 | 6.71E-16 | 65.6 | 64.8 | 2.31E-01 |
| 26 | 1.48 | 1.54 | 3.03E-16 | 67.0 | 65.8 | 7.41E-02 |
| 27 | 1.49 | 1.55 | 3.05E-17 | 69.3 | 68.4 | 1.84E-01 |
| 28 | 1.50 | 1.57 | 7.75E-18 | 70.7 | 70.7 | 9.54E-01 |
| 29 | 1.51 | 1.58 | 8.93E-19 | 70.3 | 71.1 | 2.87E-01 |
| 30 | 1.53 | 1.59 | 8.04E-18 | 71.7 | 72.2 | 5.42E-01 |
| 31 | 1.54 | 1.61 | 9.97E-17 | 72.0 | 73.2 | 1.24E-01 |
| 32 | 1.56 | 1.63 | 2.03E-17 | 71.9 | 72.7 | 3.14E-01 |
| 33 | 1.57 | 1.64 | 4.04E-16 | 73.8 | 74.7 | 2.48E-01 |
| 34 | 1.59 | 1.65 | 1.19E-15 | 74.2 | 75.9 | 4.27E-02 |
| 35 | 1.60 | 1.67 | 4.50E-15 | 74.4 | 75.4 | 2.32E-01 |
| 36 | 1.62 | 1.68 | 1.01E-14 | 74.7 | 75.3 | 5.06E-01 |
| 37 | 1.63 | 1.70 | 5.51E-15 | 73.9 | 73.5 | 6.86E-01 |
| 38 | 1.65 | 1.71 | 4.61E-16 | 73.9 | 73.2 | 4.25E-01 |
| 39 | 1.66 | 1.73 | 4.15E-17 | 75.2 | 75.7 | 6.28E-01 |
| 40 | 1.68 | 1.75 | 1.13E-16 | 74.9 | 76.3 | 1.87E-01 |
| 41 | 1.69 | 1.76 | 1.31E-15 |  |  |  |
| 42 | 1.71 | 1.78 | 2.98E-15 |  |  |  |

**Supplementary Table 2.** Least square means of daily feed intake (DFI) and body weight (BW) for the two lines of pHu animals, and significance of the line effect.

|  | DFI | | | *BW* | | |
| --- | --- | --- | --- | --- | --- | --- |
| Age | pHu- | pHu+ | *P-value* | pHu- | pHu+ | *P-value* |
| 3 | 4.7 | 4.6 | 6.99E-01 | 54.0 | 55.7 | 5.18E-02 |
| 4 | 9.0 | 9.6 | 2.92E-01 | 64.7 | 67.7 | 4.06E-03 |
| 5 | 13.7 | 12.4 | 3.57E-03 | 81.6 | 81.8 | 8.46E-01 |
| 6 | 21.1 | 18.5 | 1.20E-08 | 97.5 | 95.6 | 7.93E-02 |
| 7 | 23.9 | 21.8 | 6.06E-05 | 116.5 | 111.6 | 2.47E-04 |
| 8 | 28.4 | 26.4 | 1.26E-02 | 138.1 | 131.6 | 2.97E-05 |
| 9 | 34.6 | 34.1 | 5.54E-01 | 159.7 | 151.6 | 3.44E-06 |
| 10 | 41.0 | 39.6 | 3.63E-02 | 187.6 | 177.3 | 5.04E-07 |
| 11 | 46.4 | 45.5 | 2.31E-01 | 220.0 | 208.2 | 7.56E-07 |
| 12 | 50.4 | 50.5 | 9.15E-01 | 257.2 | 243.1 | 4.53E-07 |
| 13 | 56.2 | 56.0 | 7.43E-01 | 298.2 | 281.2 | 8.74E-08 |
| 14 | 61.4 | 61.7 | 6.60E-01 | 346.0 | 325.8 | 3.50E-08 |
| 15 | 68.3 | 68.8 | 5.58E-01 | 393.6 | 369.2 | 4.83E-09 |
| 16 | 67.1 | 68.6 | 8.71E-02 | 445.4 | 417.9 | 1.77E-09 |
| 17 | 75.6 | 76.3 | 4.97E-01 | 499.3 | 470.4 | 1.62E-08 |
| 18 | 88.0 | 88.5 | 6.10E-01 | 555.3 | 524.3 | 3.88E-08 |
| 19 | 94.1 | 94.4 | 8.48E-01 | 617.9 | 585.1 | 1.19E-07 |
| 20 | 100.3 | 101.0 | 5.12E-01 | 683.1 | 646.4 | 4.85E-08 |
| 21 | 105.8 | 108.3 | 8.36E-02 | 756.2 | 718.2 | 3.22E-07 |
| 22 | 113.4 | 114.5 | 4.65E-01 | 829.5 | 791.0 | 2.88E-06 |
| 23 | 120.2 | 123.9 | 1.35E-02 | 906.9 | 867.3 | 9.58E-06 |
| 24 | 122.7 | 125.5 | 6.47E-02 | 983.8 | 944.9 | 4.32E-05 |
| 25 | 129.1 | 132.4 | 4.03E-02 | 1063.4 | 1022.3 | 6.24E-05 |
| 26 | 136.0 | 141.0 | 5.64E-03 | 1147.3 | 1103.7 | 6.31E-05 |
| 27 | 141.1 | 147.1 | 1.43E-03 | 1235.0 | 1191.2 | 1.48E-04 |
| 28 | 144.3 | 153.6 | 6.77E-07 | 1318.8 | 1273.1 | 1.91E-04 |
| 29 | 150.3 | 157.8 | 8.96E-05 | 1409.7 | 1363.4 | 3.17E-04 |
| 30 | 153.9 | 163.6 | 2.94E-07 | 1500.8 | 1457.3 | 1.37E-03 |
| 31 | 157.6 | 166.9 | 8.49E-06 | 1586.6 | 1544.8 | 3.21E-03 |
| 32 | 161.9 | 169.4 | 7.09E-04 | 1677.5 | 1634.1 | 3.52E-03 |
| 33 | 170.5 | 177.0 | 7.74E-03 | 1769.9 | 1729.5 | 9.48E-03 |
| 34 | 172.9 | 180.6 | 4.36E-04 | 1860.4 | 1821.0 | 1.55E-02 |
| 35 | 176.0 | 181.6 | 1.31E-02 | 1955.5 | 1918.4 | 2.83E-02 |
| 36 | 178.9 | 188.9 | 4.69E-06 | 2048.4 | 2013.5 | 4.72E-02 |
| 37 | 182.4 | 190.8 | 6.67E-04 | 2141.7 | 2106.6 | 5.23E-02 |
| 38 | 182.3 | 191.3 | 2.30E-04 | 2233.9 | 2197.2 | 4.96E-02 |
| 39 | 187.9 | 195.9 | 2.74E-03 | 2325.0 | 2286.1 | 4.53E-02 |
| 40 | 193.1 | 199.3 | 2.22E-02 | 2418.0 | 2379.3 | 5.27E-02 |
| 41 | 193.1 | 200.9 | 7.86E-03 | 2518.5 | 2483.1 | 8.63E-02 |
| 42 | 200.4 | 210.2 | 2.37E-03 | 2606.7 | 2576.6 | 1.64E-01 |

**Supplementary Table 3.** Heritability estimates (+ standard errors) of the average daily gain (ADG), body weight (BW), daily cumulative feed conversion ratio (DCFCR), and daily feed intake (DFI) for the pHu+ and pHu- lines.

| Age (d) | pHu+ | | | |  | pHu- | | | |
| --- | --- | --- | --- | --- | --- | --- | --- | --- | --- |
|  | ADG | BW | DCFCR | DFI |  | ADG | BW | DCFCR | DFI |
| 3 |  |  |  |  |  |  |  |  |  |
| 4 |  | 0.32 ± 0.04 |  | 0.19 ± 0.05 |  |  | 0.54 ± 0.07 |  | 0.31 ± 0.06 |
| 5 | 0.15 ± 0.03 | 0.47 ± 0.04 | 0.19 ± 0.04 | 0.14 ± 0.03 |  | 0.30 ± 0.06 | 0.38 ± 0.07 | 0.08 ± 0.03 | 0.21 ± 0.05 |
| 6 | 0.09 ± 0.02 | 0.24 ± 0.04 | 0.12 ± 0.03 | 0.26 ± 0.04 |  | 0.28 ± 0.04 | 0.59 ± 0.05 | 0.10 ± 0.02 | 0.16 ± 0.04 |
| 7 | 0.15 ± 0.02 | 0.44 ± 0.04 | 0.09 ± 0.02 | 0.12 ± 0.03 |  | 0.37 ± 0.06 | 0.50 ± 0.06 | 0.08 ± 0.02 | 0.18 ± 0.04 |
| 8 | 0.30 ± 0.05 | 0.39 ± 0.05 | 0.10 ± 0.03 | 0.15 ± 0.05 |  | 0.32 ± 0.05 | 0.46 ± 0.05 | 0.20 ± 0.03 | 0.23 ± 0.03 |
| 9 | 0.36 ± 0.05 | 0.49 ± 0.04 | 0.10 ± 0.03 | 0.22 ± 0.04 |  | 0.53 ± 0.05 | 0.62 ± 0.04 | 0.22 ± 0.03 | 0.28 ± 0.04 |
| 10 | 0.26 ± 0.04 | 0.39 ± 0.06 | 0.08 ± 0.02 | 0.18 ± 0.03 |  | 0.45 ± 0.06 | 0.62 ± 0.05 | 0.14 ± 0.02 | 0.33 ± 0.04 |
| 11 | 0.29 ± 0.03 | 0.32 ± 0.05 | 0.13 ± 0.03 | 0.22 ± 0.03 |  | 0.43 ± 0.05 | 0.78 ± 0.04 | 0.25 ± 0.03 | 0.31 ± 0.03 |
| 12 | 0.19 ± 0.03 | 0.43 ± 0.05 | 0.12 ± 0.03 | 0.24 ± 0.04 |  | 0.57 ± 0.06 | 0.74 ± 0.06 | 0.31 ± 0.03 | 0.33 ± 0.05 |
| 13 | 0.24 ± 0.04 | 0.54 ± 0.04 | 0.28 ± 0.04 | 0.35 ± 0.04 |  | 0.24 ± 0.04 | 0.76 ± 0.04 | 0.31 ± 0.04 | 0.29 ± 0.04 |
| 14 | 0.51 ± 0.05 | 0.35 ± 0.05 | 0.24 ± 0.03 | 0.19 ± 0.04 |  | 0.46 ± 0.05 | 0.83 ± 0.02 | 0.33 ± 0.04 | 0.14 ± 0.04 |
| 15 | 0.36 ± 0.06 | 0.41 ± 0.04 | 0.23 ± 0.03 | 0.25 ± 0.04 |  | 0.43 ± 0.05 | 0.68 ± 0.04 | 0.40 ± 0.05 | 0.07 ± 0.01 |
| 16 | 0.61 ± 0.04 | 0.46 ± 0.05 | 0.28 ± 0.03 | 0.19 ± 0.03 |  | 0.35 ± 0.04 | 0.74 ± 0.03 | 0.53 ± 0.05 | 0.14 ± 0.03 |
| 17 | 0.44 ± 0.05 | 0.47 ± 0.04 | 0.29 ± 0.04 | 0.34 ± 0.03 |  | 0.41 ± 0.03 | 0.67 ± 0.03 | 0.45 ± 0.05 | 0.29 ± 0.03 |
| 18 | 0.49 ± 0.05 | 0.52 ± 0.05 | 0.32 ± 0.04 | 0.38 ± 0.04 |  | 0.44 ± 0.03 | 0.71 ± 0.03 | 0.41 ± 0.04 | 0.22 ± 0.03 |
| 19 | 0.46 ± 0.05 | 0.62 ± 0.05 | 0.37 ± 0.05 | 0.38 ± 0.04 |  | 0.39 ± 0.03 | 0.65 ± 0.05 | 0.37 ± 0.05 | 0.34 ± 0.05 |
| 20 | 0.53 ± 0.05 | 0.67 ± 0.05 | 0.47 ± 0.04 | 0.45 ± 0.04 |  | 0.50 ± 0.03 | 0.71 ± 0.03 | 0.34 ± 0.04 | 0.30 ± 0.03 |
| 21 | 0.56 ± 0.05 | 0.61 ± 0.04 | 0.34 ± 0.04 | 0.42 ± 0.05 |  | 0.53 ± 0.03 | 0.61 ± 0.05 | 0.38 ± 0.06 | 0.32 ± 0.03 |
| 22 | 0.48 ± 0.04 | 0.56 ± 0.04 | 0.41 ± 0.04 | 0.44 ± 0.04 |  | 0.43 ± 0.04 | 0.59 ± 0.03 | 0.39 ± 0.05 | 0.15 ± 0.03 |
| 23 | 0.57 ± 0.04 | 0.56 ± 0.04 | 0.44 ± 0.04 | 0.44 ± 0.04 |  | 0.46 ± 0.04 | 0.62 ± 0.03 | 0.42 ± 0.05 | 0.30 ± 0.03 |
| 24 | 0.53 ± 0.04 | 0.57 ± 0.05 | 0.47 ± 0.04 | 0.41 ± 0.04 |  | 0.40 ± 0.04 | 0.60 ± 0.03 | 0.43 ± 0.04 | 0.33 ± 0.04 |
| 25 | 0.57 ± 0.04 | 0.55 ± 0.04 | 0.40 ± 0.03 | 0.47 ± 0.04 |  | 0.36 ± 0.04 | 0.52 ± 0.03 | 0.47 ± 0.03 | 0.35 ± 0.05 |

**Supplementary Table 3 (continued).** Heritability estimates (+ standard errors) of the average daily gain (ADG), body weight (BW), daily cumulative feed conversion ratio (DCFCR), and daily feed intake (DFI) for the pHu+ and pHu- lines.

|  |  | pHu+ |  |  |  |  | pHu- |  |  |
| --- | --- | --- | --- | --- | --- | --- | --- | --- | --- |
|  | ADG | BW | DCFCR | DFI |  | ADG | BW | DCFCR | DFI |
| 26 | 0.53 ± 0.04 | 0.55 ± 0.05 | 0.49 ± 0.03 | 0.51 ± 0.04 |  | 0.53 ± 0.04 | 0.55 ± 0.03 | 0.51 ± 0.04 | 0.37 ± 0.06 |
| 27 | 0.45 ± 0.04 | 0.55 ± 0.04 | 0.36 ± 0.04 | 0.34 ± 0.03 |  | 0.42 ± 0.04 | 0.49 ± 0.03 | 0.53 ± 0.04 | 0.32 ± 0.05 |
| 28 | 0.39 ± 0.04 | 0.56 ± 0.04 | 0.34 ± 0.03 | 0.41 ± 0.04 |  | 0.35 ± 0.04 | 0.56 ± 0.03 | 0.53 ± 0.04 | 0.26 ± 0.04 |
| 29 | 0.36 ± 0.05 | 0.54 ± 0.04 | 0.33 ± 0.04 | 0.30 ± 0.05 |  | 0.34 ± 0.04 | 0.50 ± 0.03 | 0.59 ± 0.05 | 0.25 ± 0.04 |
| 30 | 0.29 ± 0.03 | 0.54 ± 0.04 | 0.34 ± 0.04 | 0.24 ± 0.03 |  | 0.26 ± 0.03 | 0.54 ± 0.04 | 0.60 ± 0.04 | 0.27 ± 0.05 |
| 31 | 0.28 ± 0.03 | 0.53 ± 0.04 | 0.32 ± 0.04 | 0.17 ± 0.03 |  | 0.25 ± 0.03 | 0.49 ± 0.04 | 0.57 ± 0.04 | 0.16 ± 0.04 |
| 32 | 0.31 ± 0.04 | 0.53 ± 0.04 | 0.28 ± 0.04 | 0.23 ± 0.03 |  | 0.25 ± 0.04 | 0.51 ± 0.04 | 0.51 ± 0.04 | 0.28 ± 0.04 |
| 33 | 0.35 ± 0.04 | 0.51 ± 0.04 | 0.31 ± 0.03 | 0.21 ± 0.02 |  | 0.37 ± 0.04 | 0.50 ± 0.03 | 0.54 ± 0.04 | 0.20 ± 0.04 |
| 34 | 0.36 ± 0.04 | 0.51 ± 0.04 | 0.30 ± 0.04 | 0.26 ± 0.03 |  | 0.31 ± 0.04 | 0.47 ± 0.04 | 0.58 ± 0.04 | 0.32 ± 0.03 |
| 35 | 0.36 ± 0.04 | 0.52 ± 0.04 | 0.30 ± 0.03 | 0.33 ± 0.04 |  | 0.30 ± 0.04 | 0.49 ± 0.04 | 0.61 ± 0.04 | 0.22 ± 0.03 |
| 36 | 0.35 ± 0.04 | 0.51 ± 0.04 | 0.30 ± 0.03 | 0.17 ± 0.03 |  | 0.25 ± 0.03 | 0.43 ± 0.03 | 0.57 ± 0.04 | 0.20 ± 0.04 |
| 37 | 0.29 ± 0.04 | 0.49 ± 0.04 | 0.28 ± 0.04 | 0.13 ± 0.02 |  | 0.12 ± 0.03 | 0.42 ± 0.04 | 0.56 ± 0.05 | 0.16 ± 0.04 |
| 38 | 0.26 ± 0.04 | 0.47 ± 0.03 | 0.27 ± 0.04 | 0.22 ± 0.03 |  | 0.14 ± 0.02 | 0.41 ± 0.04 | 0.57 ± 0.04 | 0.16 ± 0.03 |
| 39 | 0.29 ± 0.03 | 0.45 ± 0.03 | 0.33 ± 0.05 | 0.25 ± 0.03 |  | 0.23 ± 0.04 | 0.38 ± 0.04 | 0.59 ± 0.04 | 0.30 ± 0.04 |
| 40 | 0.23 ± 0.03 | 0.43 ± 0.04 | 0.34 ± 0.05 | 0.16 ± 0.03 |  | 0.21 ± 0.05 | 0.38 ± 0.04 | 0.54 ± 0.03 | 0.24 ± 0.03 |
| 41 |  | 0.42 ± 0.03 | 0.36 ± 0.06 | 0.18 ± 0.02 |  |  | 0.37 ± 0.03 | 0.52 ± 0.04 | 0.24 ± 0.04 |
| 42 |  | 0.39 ± 0.03 | 0.36 ± 0.08 | 0.11 ± 0.03 |  |  | 0.35 ± 0.03 | 0.63 ± 0.07 | 0.24 ± 0.03 |

**Supplementary Table 4.** Genetic correlations (+standard errors) between final cumulative feed conversion ratio (DCFCR_42_) and average daily gain (ADG), body weight (BW), daily cumulative feed conversion ratio (DCFCR), and daily feed intake (DFI).

|  | pHu+ | | |  |  |  | pHu- | | |
| --- | --- | --- | --- | --- | --- | --- | --- | --- | --- |
|  | ADG | BW | DCFCR | DFI |  | ADG | BW | DCFCR | DFI |
| 3 |  |  |  |  |  |  |  |  |  |
| 4 |  | 0.40 ± 0.07 |  | -0.03 ± 0.07 |  |  | -0.21 ± 0.11 |  | 0.07 ± 0.13 |
| 5 | -0.25 ± 0.07 | 0.04 ± 0.06 | 0.53 ± 0.14 | 0.71 ± 0.09 |  | 0.14 ± 0.10 | 0.10 ± 0.06 | 0.24 ± 0.09 | 0.28 ± 0.08 |
| 6 | 0.05 ± 0.07 | 0.06 ± 0.06 | 0.17 ± 0.10 | 0.66 ± 0.12 |  | 0.05 ± 0.14 | 0.00 ± 0.11 | 0.46 ± 0.11 | 0.21 ± 0.08 |
| 7 | 0.40 ± 0.05 | 0.21 ± 0.05 | 0.10 ± 0.07 | 0.61 ± 0.07 |  | -0.12 ± 0.11 | -0.01 ± 0.05 | 0.88 ± 0.10 | 0.72 ± 0.09 |
| 8 | 0.22 ± 0.07 | 0.06 ± 0.05 | 0.08 ± 0.04 | 0.76 ± 0.06 |  | 0.08 ± 0.08 | 0.03 ± 0.07 | 0.86 ± 0.11 | 0.70 ± 0.07 |
| 9 | 0.12 ± 0.06 | 0.30 ± 0.05 | -0.16 ± 0.04 | 0.69 ± 0.08 |  | -0.04 ± 0.10 | -0.12 ± 0.07 | 0.84 ± 0.11 | 0.51 ± 0.09 |
| 10 | 0.27 ± 0.06 | 0.39 ± 0.05 | 0.34 ± 0.06 | 0.52 ± 0.07 |  | 0.02 ± 0.09 | 0.07 ± 0.10 | 0.75 ± 0.11 | 0.24 ± 0.10 |
| 11 | -0.05 ± 0.06 | 0.13 ± 0.06 | 0.71 ± 0.05 | 0.57 ± 0.05 |  | 0.13 ± 0.11 | 0.03 ± 0.09 | 0.80 ± 0.12 | 0.23 ± 0.06 |
| 12 | 0.14 ± 0.05 | 0.26 ± 0.06 | 0.53 ± 0.06 | 0.70 ± 0.07 |  | -0.02 ± 0.10 | -0.06 ± 0.09 | 0.74 ± 0.09 | 0.39 ± 0.06 |
| 13 | 0.16 ± 0.06 | 0.01 ± 0.06 | 0.65 ± 0.07 | 0.71 ± 0.05 |  | 0.04 ± 0.07 | -0.05 ± 0.08 | 0.69 ± 0.09 | 0.46 ± 0.05 |
| 14 | 0.06 ± 0.06 | 0.03 ± 0.05 | 0.65 ± 0.06 | 0.59 ± 0.10 |  | -0.20 ± 0.06 | 0.01 ± 0.09 | 0.66 ± 0.07 | 0.29 ± 0.10 |
| 15 | 0.25 ± 0.06 | 0.10 ± 0.07 | 0.71 ± 0.05 | 0.45 ± 0.15 |  | -0.32 ± 0.06 | -0.08 ± 0.08 | 0.55 ± 0.06 | 0.21 ± 0.09 |
| 16 | 0.32 ± 0.08 | 0.14 ± 0.05 | 0.64 ± 0.05 | 0.25 ± 0.12 |  | -0.03 ± 0.06 | -0.09 ± 0.08 | 0.66 ± 0.04 | 0.25 ± 0.09 |
| 17 | 0.24 ± 0.06 | 0.08 ± 0.05 | 0.55 ± 0.05 | 0.46 ± 0.08 |  | 0.03 ± 0.07 | -0.08 ± 0.07 | 0.71 ± 0.06 | 0.13 ± 0.06 |
| 18 | 0.08 ± 0.07 | 0.15 ± 0.05 | 0.59 ± 0.05 | 0.41 ± 0.10 |  | 0.22 ± 0.08 | -0.01 ± 0.06 | 0.70 ± 0.07 | 0.05 ± 0.08 |
| 19 | 0.15 ± 0.07 | 0.11 ± 0.05 | 0.76 ± 0.04 | 0.33 ± 0.16 |  | 0.22 ± 0.09 | -0.02 ± 0.05 | 0.78 ± 0.04 | 0.18 ± 0.07 |
| 20 | 0.11 ± 0.06 | 0.03 ± 0.05 | 0.79 ± 0.06 | 0.28 ± 0.08 |  | 0.12 ± 0.09 | 0.04 ± 0.06 | 0.71 ± 0.05 | 0.29 ± 0.06 |
| 21 | 0.10 ± 0.06 | 0.00 ± 0.06 | 0.71 ± 0.06 | 0.41 ± 0.10 |  | 0.17 ± 0.09 | 0.04 ± 0.06 | 0.62 ± 0.07 | 0.21 ± 0.08 |
| 22 | 0.29 ± 0.07 | 0.13 ± 0.06 | 0.63 ± 0.04 | 0.55 ± 0.14 |  | 0.01 ± 0.06 | 0.06 ± 0.06 | 0.70 ± 0.06 | 0.15 ± 0.06 |
| 23 | 0.22 ± 0.06 | 0.20 ± 0.07 | 0.68 ± 0.05 | 0.37 ± 0.08 |  | 0.11 ± 0.07 | 0.07 ± 0.05 | 0.68 ± 0.07 | 0.46 ± 0.09 |
| 24 | 0.24 ± 0.07 | 0.21 ± 0.07 | 0.64 ± 0.04 | 0.36 ± 0.08 |  | 0.14 ± 0.06 | 0.04 ± 0.07 | 0.82 ± 0.06 | 0.46 ± 0.08 |
| 25 | 0.20 ± 0.07 | 0.30 ± 0.07 | 0.50 ± 0.04 | 0.37 ± 0.07 |  | 0.02 ± 0.06 | 0.09 ± 0.07 | 0.80 ± 0.08 | 0.57 ± 0.09 |

**Supplementary Table 4 (continued).** Genetic correlations (+standard errors) between final cumulative feed conversion ratio (DCFCR_42_) and average daily gain (ADG), body weight (BW), daily cumulative feed conversion ratio (DCFCR), and daily feed intake (DFI).

|  | pHu+ | | |  |  |  | pHu- | | |
| --- | --- | --- | --- | --- | --- | --- | --- | --- | --- |
|  | ADG | BW | DCFCR | DFI |  | ADG | BW | DCFCR | DFI |
| 26 | 0.18 ± 0.06 | 0.27 ± 0.06 | 0.51 ± 0.03 | 0.33 ± 0.08 |  | 0.17 ± 0.06 | 0.09 ± 0.09 | 0.83 ± 0.06 | 0.66 ± 0.07 |
| 27 | 0.07 ± 0.07 | 0.27 ± 0.06 | 0.49 ± 0.03 | 0.25 ± 0.07 |  | 0.08 ± 0.06 | 0.06 ± 0.09 | 0.86 ± 0.07 | 0.66 ± 0.09 |
| 28 | 0.04 ± 0.09 | 0.25 ± 0.06 | 0.53 ± 0.03 | 0.50 ± 0.06 |  | -0.14 ± 0.06 | 0.07 ± 0.08 | 0.85 ± 0.07 | 0.72 ± 0.07 |
| 29 | 0.03 ± 0.08 | 0.25 ± 0.06 | 0.66 ± 0.04 | 0.50 ± 0.09 |  | -0.11 ± 0.06 | 0.08 ± 0.07 | 0.86 ± 0.06 | 0.51 ± 0.09 |
| 30 | 0.13 ± 0.08 | 0.22 ± 0.08 | 0.68 ± 0.02 | 0.31 ± 0.08 |  | -0.23 ± 0.12 | 0.02 ± 0.06 | 0.92 ± 0.06 | 0.66 ± 0.10 |
| 31 | 0.06 ± 0.08 | 0.22 ± 0.08 | 0.77 ± 0.02 | 0.43 ± 0.11 |  | -0.22 ± 0.07 | 0.03 ± 0.06 | 0.91 ± 0.04 | 0.50 ± 0.12 |
| 32 | -0.02 ± 0.09 | 0.22 ± 0.08 | 0.76 ± 0.02 | 0.27 ± 0.12 |  | -0.23 ± 0.09 | 0.00 ± 0.06 | 0.92 ± 0.06 | 0.36 ± 0.09 |
| 33 | 0.12 ± 0.07 | 0.21 ± 0.09 | 0.73 ± 0.02 | 0.28 ± 0.12 |  | -0.37 ± 0.08 | -0.01 ± 0.06 | 0.91 ± 0.05 | 0.46 ± 0.09 |
| 34 | -0.02 ± 0.09 | 0.19 ± 0.10 | 0.82 ± 0.01 | 0.64 ± 0.09 |  | -0.27 ± 0.09 | -0.06 ± 0.06 | 0.94 ± 0.04 | 0.52 ± 0.07 |
| 35 | 0.04 ± 0.08 | 0.17 ± 0.10 | 0.81 ± 0.01 | 0.40 ± 0.08 |  | -0.32 ± 0.09 | -0.09 ± 0.08 | 0.94 ± 0.04 | 0.40 ± 0.07 |
| 36 | 0.10 ± 0.08 | 0.15 ± 0.12 | 0.81 ± 0.01 | 0.34 ± 0.09 |  | -0.30 ± 0.12 | -0.09 ± 0.07 | 0.96 ± 0.04 | 0.53 ± 0.14 |
| 37 | -0.09 ± 0.13 | 0.18 ± 0.09 | 0.88 ± 0.01 | 0.57 ± 0.09 |  | -0.61 ± 0.09 | -0.11 ± 0.08 | 0.97 ± 0.03 | 0.55 ± 0.09 |
| 38 | 0.08 ± 0.13 | 0.17 ± 0.12 | 0.93 ± 0.01 | 0.44 ± 0.09 |  | -0.44 ± 0.09 | -0.14 ± 0.07 | 0.98 ± 0.03 | 0.34 ± 0.07 |
| 39 | -0.12 ± 0.07 | 0.16 ± 0.12 | 0.95 ± 0.01 | 0.57 ± 0.09 |  | -0.28 ± 0.08 | -0.16 ± 0.06 | 0.99 ± 0.02 | 0.25 ± 0.07 |
| 40 | -0.37 ± 0.08 | 0.17 ± 0.10 | 0.98 ± 0.01 | 0.70 ± 0.07 |  | -0.59 ± 0.11 | -0.12 ± 0.07 | 0.99 ± 0.01 | 0.41 ± 0.08 |
| 41 |  | 0.17 ± 0.09 | 0.98 ± 0.01 | 0.15 ± 0.07 |  |  | -0.12 ± 0.08 | 0.99 ± 0.01 | 0.18 ± 0.08 |
| 42 |  | 0.16 ± 0.09 | 1.00 ± 0.01 | 0.41 ± 0.10 |  |  | -0.13 ± 0.08 | 1.00 ± 0.02 | 0.26 ± 0.12 |

**Supplementary Table 5.** Genetic correlations (+standard errors) between pHu and average daily gain (ADG), body weight (BW), daily cumulative feed conversion ratio (DCFCR), and daily feed intake (DFI).

|  | pHu+ | | |  | | | pHu- | | |
| --- | --- | --- | --- | --- | --- | --- | --- | --- | --- |
|  | ADG | BW | CFCR | DFI |  | ADG | BW | CFCR | DFI |
| 3 |  |  |  |  |  |  |  |  |  |
| 4 |  | -0.21 ± 0.06 |  | 0.38 ± 0.10 |  |  | 0.24 ± 0.08 |  | -0.01 ± 0.12 |
| 5 | 0.33 ± 0.07 | 0.07 ± 0.09 | -0.25 ± 0.19 | -0.45 ± 0.08 |  | 0.07 ± 0.09 | 0.13 ± 0.05 | 0.04 ± 0.12 | 0.12 ± 0.12 |
| 6 | -0.16 ± 0.07 | -0.02 ± 0.07 | 0.05 ± 0.13 | -0.52 ± 0.08 |  | -0.06 ± 0.17 | 0.12 ± 0.11 | 0.49 ± 0.12 | 0.30 ± 0.08 |
| 7 | -0.59 ± 0.06 | -0.26 ± 0.07 | 0.17 ± 0.12 | -0.41 ± 0.11 |  | 0.01 ± 0.09 | 0.03 ± 0.06 | 0.72 ± 0.12 | 0.41 ± 0.10 |
| 8 | -0.38 ± 0.08 | -0.13 ± 0.08 | 0.18 ± 0.06 | -0.81 ± 0.07 |  | 0.03 ± 0.11 | 0.01 ± 0.06 | 0.86 ± 0.12 | 0.60 ± 0.09 |
| 9 | -0.37 ± 0.07 | -0.47 ± 0.07 | 0.38 ± 0.05 | -0.66 ± 0.11 |  | 0.10 ± 0.08 | 0.05 ± 0.06 | 0.77 ± 0.11 | 0.32 ± 0.09 |
| 10 | -0.25 ± 0.07 | -0.47 ± 0.08 | -0.12 ± 0.04 | -0.31 ± 0.08 |  | 0.17 ± 0.12 | 0.06 ± 0.08 | 0.86 ± 0.13 | 0.27 ± 0.11 |
| 11 | 0.07 ± 0.06 | -0.27 ± 0.06 | -0.45 ± 0.08 | -0.48 ± 0.11 |  | 0.17 ± 0.13 | 0.13 ± 0.09 | 0.75 ± 0.18 | 0.01 ± 0.09 |
| 12 | -0.20 ± 0.07 | -0.33 ± 0.08 | -0.46 ± 0.13 | -0.66 ± 0.09 |  | 0.02 ± 0.09 | 0.08 ± 0.09 | 0.67 ± 0.11 | 0.25 ± 0.09 |
| 13 | -0.22 ± 0.07 | 0.00 ± 0.07 | -0.63 ± 0.11 | -0.71 ± 0.09 |  | -0.05 ± 0.06 | 0.08 ± 0.08 | 0.61 ± 0.10 | 0.27 ± 0.06 |
| 14 | -0.14 ± 0.05 | -0.05 ± 0.08 | -0.53 ± 0.08 | -0.47 ± 0.07 |  | -0.17 ± 0.06 | 0.02 ± 0.09 | 0.75 ± 0.07 | 0.46 ± 0.13 |
| 15 | -0.32 ± 0.06 | -0.14 ± 0.07 | -0.55 ± 0.06 | -0.23 ± 0.18 |  | -0.26 ± 0.06 | -0.05 ± 0.06 | 0.44 ± 0.10 | 0.09 ± 0.16 |
| 16 | -0.38 ± 0.07 | -0.17 ± 0.07 | -0.49 ± 0.05 | -0.07 ± 0.14 |  | 0.17 ± 0.07 | -0.06 ± 0.08 | 0.54 ± 0.06 | 0.09 ± 0.12 |
| 17 | -0.27 ± 0.05 | -0.10 ± 0.07 | -0.33 ± 0.09 | -0.43 ± 0.07 |  | 0.17 ± 0.07 | -0.06 ± 0.07 | 0.50 ± 0.08 | 0.20 ± 0.05 |
| 18 | -0.13 ± 0.06 | -0.19 ± 0.06 | -0.32 ± 0.12 | -0.35 ± 0.07 |  | 0.42 ± 0.07 | 0.04 ± 0.07 | 0.47 ± 0.09 | 0.26 ± 0.09 |
| 19 | -0.21 ± 0.06 | -0.15 ± 0.06 | -0.56 ± 0.12 | -0.26 ± 0.05 |  | 0.38 ± 0.06 | 0.03 ± 0.07 | 0.58 ± 0.07 | 0.45 ± 0.06 |
| 20 | -0.17 ± 0.05 | -0.07 ± 0.06 | -0.56 ± 0.13 | -0.28 ± 0.08 |  | 0.17 ± 0.06 | 0.08 ± 0.07 | 0.58 ± 0.09 | 0.29 ± 0.07 |
| 21 | -0.19 ± 0.06 | -0.07 ± 0.06 | -0.45 ± 0.11 | -0.44 ± 0.10 |  | 0.23 ± 0.07 | 0.10 ± 0.06 | 0.44 ± 0.12 | 0.23 ± 0.06 |
| 22 | -0.37 ± 0.06 | -0.22 ± 0.05 | -0.38 ± 0.09 | -0.48 ± 0.11 |  | 0.06 ± 0.06 | 0.14 ± 0.05 | 0.49 ± 0.11 | 0.02 ± 0.07 |
| 23 | -0.25 ± 0.06 | -0.28 ± 0.05 | -0.43 ± 0.13 | -0.29 ± 0.09 |  | 0.20 ± 0.05 | 0.16 ± 0.05 | 0.48 ± 0.11 | 0.45 ± 0.09 |
| 24 | -0.33 ± 0.06 | -0.31 ± 0.05 | -0.36 ± 0.12 | -0.31 ± 0.08 |  | 0.34 ± 0.05 | 0.11 ± 0.05 | 0.55 ± 0.10 | 0.40 ± 0.07 |
| 25 | -0.31 ± 0.05 | -0.38 ± 0.05 | -0.21 ± 0.10 | -0.41 ± 0.08 |  | 0.26 ± 0.05 | 0.19 ± 0.04 | 0.44 ± 0.11 | 0.53 ± 0.07 |

**Supplementary Table 5 (continued).** Genetic correlations (+standard errors) between pHu and average daily gain (ADG), body weight (BW), daily cumulative feed conversion ratio (DCFCR), and daily feed intake (DFI).

|  | pHu+ | | |  | | | pHu- | | |
| --- | --- | --- | --- | --- | --- | --- | --- | --- | --- |
|  | ADG | BW | CFCR | DFI |  | ADG | BW | CFCR | DFI |
| 26 | -0.24 ± 0.05 | -0.36 ± 0.05 | -0.21 ± 0.10 | -0.24 ± 0.07 |  | 0.37 ± 0.05 | 0.23 ± 0.06 | 0.47 ± 0.08 | 0.66 ± 0.09 |
| 27 | -0.15 ± 0.06 | -0.37 ± 0.05 | -0.15 ± 0.09 | -0.16 ± 0.10 |  | 0.22 ± 0.06 | 0.21 ± 0.05 | 0.47 ± 0.11 | 0.48 ± 0.10 |
| 28 | -0.07 ± 0.07 | -0.35 ± 0.05 | -0.17 ± 0.09 | -0.43 ± 0.12 |  | 0.01 ± 0.06 | 0.22 ± 0.05 | 0.43 ± 0.13 | 0.46 ± 0.07 |
| 29 | 0.01 ± 0.07 | -0.34 ± 0.05 | -0.38 ± 0.12 | -0.49 ± 0.09 |  | 0.09 ± 0.06 | 0.23 ± 0.05 | 0.46 ± 0.12 | 0.45 ± 0.09 |
| 30 | -0.15 ± 0.07 | -0.32 ± 0.05 | -0.38 ± 0.07 | -0.21 ± 0.07 |  | 0.11 ± 0.13 | 0.19 ± 0.05 | 0.49 ± 0.14 | 0.63 ± 0.11 |
| 31 | -0.10 ± 0.08 | -0.29 ± 0.05 | -0.51 ± 0.09 | -0.35 ± 0.09 |  | 0.09 ± 0.07 | 0.21 ± 0.05 | 0.48 ± 0.11 | 0.42 ± 0.14 |
| 32 | 0.09 ± 0.08 | -0.30 ± 0.05 | -0.51 ± 0.09 | -0.27 ± 0.07 |  | 0.17 ± 0.07 | 0.21 ± 0.05 | 0.48 ± 0.13 | 0.44 ± 0.10 |
| 33 | -0.13 ± 0.08 | -0.30 ± 0.05 | -0.41 ± 0.11 | -0.11 ± 0.09 |  | -0.11 ± 0.07 | 0.20 ± 0.05 | 0.53 ± 0.13 | 0.53 ± 0.11 |
| 34 | 0.03 ± 0.08 | -0.26 ± 0.06 | -0.60 ± 0.08 | -0.58 ± 0.08 |  | -0.01 ± 0.08 | 0.19 ± 0.05 | 0.51 ± 0.10 | 0.50 ± 0.06 |
| 35 | 0.10 ± 0.08 | -0.25 ± 0.07 | -0.56 ± 0.09 | -0.25 ± 0.08 |  | -0.07 ± 0.08 | 0.14 ± 0.06 | 0.53 ± 0.11 | 0.37 ± 0.11 |
| 36 | -0.02 ± 0.08 | -0.24 ± 0.07 | -0.51 ± 0.11 | -0.11 ± 0.10 |  | -0.06 ± 0.10 | 0.14 ± 0.06 | 0.57 ± 0.11 | 0.45 ± 0.18 |
| 37 | 0.24 ± 0.14 | -0.24 ± 0.06 | -0.64 ± 0.11 | -0.45 ± 0.09 |  | -0.23 ± 0.09 | 0.11 ± 0.07 | 0.60 ± 0.11 | 0.47 ± 0.12 |
| 38 | 0.11 ± 0.12 | -0.22 ± 0.08 | -0.73 ± 0.10 | -0.42 ± 0.10 |  | -0.24 ± 0.08 | 0.10 ± 0.06 | 0.56 ± 0.09 | 0.21 ± 0.10 |
| 39 | 0.25 ± 0.10 | -0.20 ± 0.06 | -0.78 ± 0.10 | -0.63 ± 0.08 |  | -0.09 ± 0.07 | 0.07 ± 0.06 | 0.55 ± 0.09 | 0.13 ± 0.06 |
| 40 | 0.58 ± 0.08 | -0.19 ± 0.06 | -0.82 ± 0.10 | -0.77 ± 0.08 |  | -0.56 ± 0.10 | 0.07 ± 0.06 | 0.55 ± 0.07 | 0.23 ± 0.08 |
| 41 |  | -0.18 ± 0.06 | -0.86 ± 0.08 | 0.05 ± 0.09 |  |  | 0.06 ± 0.06 | 0.59 ± 0.05 | 0.02 ± 0.08 |
| 42 |  | -0.14 ± 0.06 | -0.89 ± 0.06 | -0.45 ± 0.08 |  |  | 0.04 ± 0.06 | 0.71 ± 0.05 | 0.24 ± 0.17 |

**Supplementary Table 6;** Genetic correlations (+standard errors) between BMY and daily cumulative feed conversion ratio (DCFCR)

| Age (d) | pHu+ | pHu- |  | Age (d) | pHu+ | pHu- |
| --- | --- | --- | --- | --- | --- | --- |
| 5 | 0.24 ± 0.19 | 0.00 ± 0.14 |  | 24 | 0.56 ± 0.07 | 0.11 ± 0.06 |
| 6 | 0.26 ± 0.16 | 0.03 ± 0.12 |  | 25 | 0.47 ± 0.07 | 0.10 ± 0.07 |
| 7 | 0.35 ± 0.16 | -0.04 ± 0.13 |  | 26 | 0.46 ± 0.06 | 0.11 ± 0.07 |
| 8 | 0.33 ± 0.07 | 0.21 ± 0.12 |  | 27 | 0.63 ± 0.06 | 0.12 ± 0.07 |
| 9 | 0.42 ± 0.07 | 0.22 ± 0.12 |  | 28 | 0.62 ± 0.06 | 0.09 ± 0.09 |
| 10 | 0.31 ± 0.07 | 0.22 ± 0.13 |  | 29 | 0.54 ± 0.07 | 0.08 ± 0.07 |
| 11 | 0.30 ± 0.06 | 0.28 ± 0.12 |  | 30 | 0.51 ± 0.06 | 0.09 ± 0.09 |
| 12 | 0.21 ± 0.08 | 0.39 ± 0.09 |  | 31 | 0.42 ± 0.07 | 0.08 ± 0.12 |
| 13 | 0.11 ± 0.08 | 0.32 ± 0.10 |  | 32 | 0.44 ± 0.07 | 0.04 ± 0.13 |
| 14 | 0.21 ± 0.08 | 0.27 ± 0.09 |  | 33 | 0.55 ± 0.07 | 0.04 ± 0.10 |
| 15 | 0.39 ± 0.06 | 0.21 ± 0.09 |  | 34 | 0.36 ± 0.08 | 0.07 ± 0.11 |
| 16 | 0.20 ± 0.05 | 0.21 ± 0.09 |  | 35 | 0.40 ± 0.08 | 0.01 ± 0.13 |
| 17 | 0.45 ± 0.06 | 0.27 ± 0.09 |  | 36 | 0.35 ± 0.08 | 0.04 ± 0.12 |
| 18 | 0.60 ± 0.05 | 0.29 ± 0.06 |  | 37 | 0.31 ± 0.07 | 0.06 ± 0.13 |
| 19 | 0.38 ± 0.06 | 0.23 ± 0.06 |  | 38 | 0.26 ± 0.08 | 0.05 ± 0.13 |
| 20 | 0.46 ± 0.06 | 0.28 ± 0.07 |  | 39 | 0.24 ± 0.09 | 0.06 ± 0.14 |
| 21 | 0.49 ± 0.06 | 0.20 ± 0.09 |  | 40 | 0.17 ± 0.09 | 0.04 ± 0.12 |
| 22 | 0.51 ± 0.06 | 0.12 ± 0.08 |  | 41 | 0.09 ± 0.07 | 0.06 ± 0.11 |
| 23 | 0.48 ± 0.07 | 0.10 ± 0.08 |  | 42 | 0.03 ± 0.03 | 0.07 ± 0.10 |
